# Adolescents hospitalized in adult psychiatric departments: socio-demographic features and clinical profile

**DOI:** 10.1192/j.eurpsy.2025.1152

**Published:** 2025-08-26

**Authors:** A. Karmous, H. Ghabi, M. Karoui, H. Nefzi, R. Kammoun, F. Ellouze

**Affiliations:** 1 emergency and outpatient department; 2 Department G, Razi hospital, Manouba, Tunisia

## Abstract

**Introduction:**

Adolescence is a critical period of physical, cognitive and emotional development. Mental health among adolescents is a major public health issue. The number of teenagers requiring psychiatric care is constantly increasing and several studies have found high prevalences of psychiatric disorders in this group.

**Objectives:**

The aim of the study was to describe sociodemographic and clinical characteristics of adolescents hospitalized in an adult psychiatric department.

**Methods:**

We carried out a retrospective descriptive study. It included adolescent patients, aged between 16 and 19 years, who were admitted to the department G of Razi Hospital (Tunisia), between May 2019 and May 2024 (the minimum age for hospitalization in adult psychiatric wards was 16). Data were collected from patients’ files.

**Results:**

Thirty-five adolescents were included. The mean age was 17.8 ± 0.8 years. A male predominance was noted with 65.7% of patients. We found that 57.1% of teenagers were using at least one psychoactive substance. Tobacco was the most used substance (54.3%), followed by cannabis (42.9%) and alcohol (40%). The most common motive for hospitalization was behavioral disturbances with 79.1% of patients. The most common psychiatric disorders were schizophrenia (32.6%) and schizophreniform disorder (18.6%). Antipsychotics were the most prescribed medication (58.13%) followed by benzodiazepines (30%).

**Conclusions:**

Knowing the profile of adolescents hospitalized in psychiatry, their sociodemographic and clinical characteristics would enable us to better the care we offer to them.

**Disclosure of Interest:**

None Declared

